# Presurgical orthodontic treatment with ultraearly application of premaxilla-guiding nasoalveolar molding in feeding neonates: A case series

**DOI:** 10.1016/j.jfscie.2025.100055

**Published:** 2025-09-25

**Authors:** Keiichiro Watanabe, Kaoru Yoshinaga, Sooha Matsuki, Shunsuke Mima, Hiroyuki Yamasaki, Kazuhide Mineda, Masashi Suzue, Ryuji Nakagawa, Kenichi Suga, Takashi Kaji, Ichiro Hashimoto, Eiji Tanaka

**Affiliations:** a Department of Orthodontics and Dentofacial Orthopedics, Tokushima University Graduate School of Biomedical Sciences, Tokushima, Japan; b Department of Plastic and Reconstructive Surgery, Tokushima University Graduate School of Biomedical Sciences, Tokushima, Japan; c Department of Pediatrics, Tokushima University Hospital, Tokushima, Japan; d Department of Women’s Health, Tokushima University Graduate School of Biomedical Sciences, Tokushima, Japan

**Keywords:** Cleft lip and palate, presurgical orthodontic treatment, nasoalveolar molding

## Abstract

**Background:**

The objective of this study was to present 3 cases of bilateral cleft lip and palate in which early application of premaxilla-guiding nasoalveolar molding (PG-NAM) achieved proper premaxillary alignment, columella, and upper lip lengthening by 2 months of age.

**Methods:**

Patients with bilateral cleft lip and palate were referred shortly after birth for intraoral scanning, which enables the creation of a 3-dimensional–printed jaw model. Using the jaw model, the Hotz plate was designed as a feeding plate. One week after birth, a nasal stent was applied to the Hotz plate. Once the stent was in place, 2 elastic lip tapes were used to prevent premaxillary collapse.

**Results:**

In all 3 cases, premaxillary alignment was completed within 1 through 2 months of appliance placement, and nasal septal curvature was observed after posterior premaxillary movement; however, at the time of palatoplasty, the nasal septum was straightened in all cases.

**Conclusions:**

These findings suggest ultraearly application of PG-NAM achieves appropriate premaxillary alignment, columella, and upper lip lengthening by 2 months of age, allowing for smooth surgical interventions.

## Graphical abstract

**Figure:**
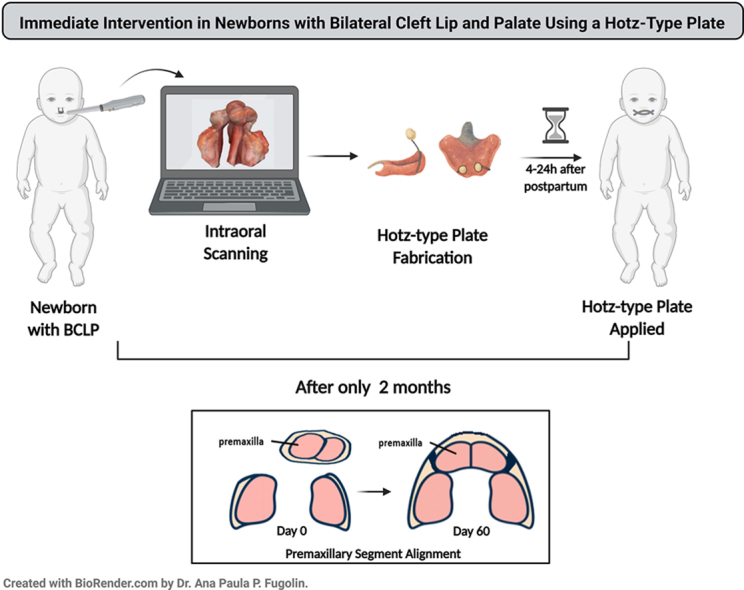

**Why Is This Important?**

Neonates with bilateral cleft lip and palate often show a protrusive premaxilla. In cases with a protruding premaxilla, early application of a feeding plate allows for proper premaxillary alignment, columellar lengthening, and upper lip stretching. In this study, the authors introduce this method as premaxilla-guiding nasoalveolar molding (PG-NAM). Early application of PG-NAM achieves nonsurgical correction of premaxillary alignment, nasal morphology, and reduction of palatal and alveolar cleft widths, facilitating lip and palatal surgeries. The implementation of this method requires the collaboration of the multidisciplinary team members in a cleft lip and palate center. In this center, the obstetricians inform the team members of the expected delivery dates for prenatally diagnosed cases. Informed consent for PG-NAM is obtained from the parents before birth, allowing ultraearly oral impressions to be taken by an oral scanner within 30 minutes of birth. This enables early placement of the feeding plate, which facilitates rapid premaxillary alignment without collapse. The ultraearly application of PG-NAM within 4 through 24 hours after birth might be a feasible remedy to achieve stable premaxillary alignment.

## Introduction

Cleft lip and palate (CLP) is the most common structural abnormality in the craniofacial region, with an incidence of approximately 1 per 500 through 700 births.^1^^,^^2^ Patients with CLP often have wide cleft widths at birth, both in the alveolar bone and palate. Performing surgery without addressing these dimensions may lead to complications such as scar formation because of overstretching of the mucosa during palatoplasty, which can negatively affect the prognosis of the maxilla after secondary bone grafting.^3^^,^^4^ The use of a Hotz appliance to reduce the width of the palatal cleft minimizes periosteal exposure in the palate and allows surgical techniques that mitigate adverse effects on maxillary growth during development.^5^^,^^6^ Decrease in the alveolar cleft width enhances the likelihood of sufficient bone graft survival after secondary bone grafting and facilitates normal tooth eruption and movement within the cleft area.^6^^,^^7^ Thus, cleft reduction has a substantial impact on their subsequent growth and treatment complexity. However, delayed initiation of maxillary alignment or improper use of the Hotz appliance may result in inadequate cleft reduction.^8^^,^^9^

In addition, nasal stent-equipped Hotz appliances or nasoalveolar molding (NAM) enable correction of nasal tip asymmetry through columellar lengthening.^10^^,^^11^ Surgical extension of the columella often fails to provide stable lengthening, especially in bilateral CLP (BCLP) cases, so early columellar lengthening during maxillary guidance is beneficial.^11^^,^^12^ Effective lengthening of the columella and correction of the nasal tip is limited to the first 3 months of life.^12^

In neonates with BCLP, the premaxilla often protrudes forward. Accurate presurgical alignment of the premaxilla using a Hotz appliance or lip tape (band) is crucial for favorable growth and optimal postsurgical lip morphology.^13^ In cases with a protruding premaxilla, the early application of a Hotz-type feeding plate and the use of 2 elastic lip tapes (1 to close the lips, 1 to prevent premaxillary drooping) allow for proper premaxillary alignment, columellar lengthening, and nasal tip projection in infants with BCLP.^7^ We refer to this method as premaxilla-guiding NAM (PG-NAM). Early application of PG-NAM achieves nonsurgical correction of premaxillary alignment, nasal morphology, and reduction of palatal and alveolar cleft widths, facilitating lip and palatal surgeries.^5^

In our report, the objective was to present 3 cases of BCLP treated with an ultraearly application of PG-NAM and to explore the effectiveness of this treatment approach for neonates with BCLP.

## Methods

### Model data acquisition for PG-NAM fabrication

**Figure 1:**
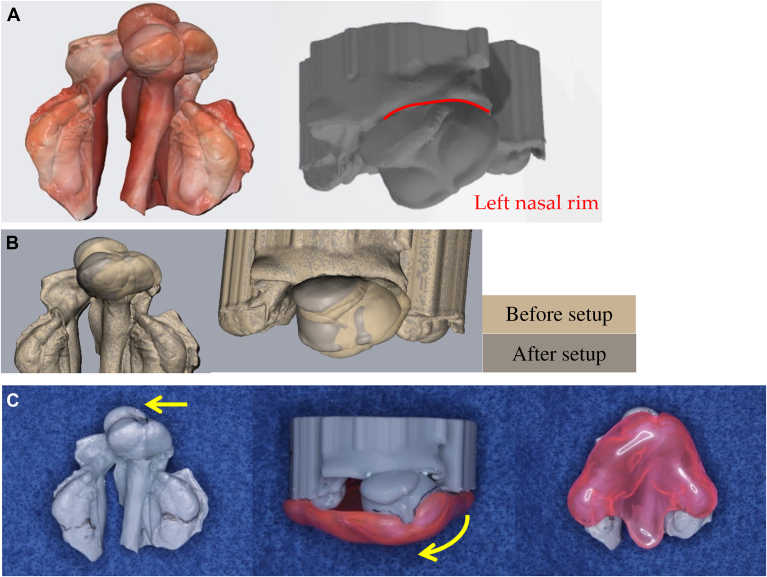
Basic setup for staged fabrication of the Hotz-type feeding plate in the treatment of bilateral cleft lip and palate. **A.** Intraoral scan obtained 30 minutes postnatally (occlusal view) and corresponding 3-dimensional model (frontal view). **B.** Superimposition of before setup (gray) and after setup (brown) simulation models for premaxillary alignment (occlusal and frontal views). **C.** Three-dimensional-printed setup model and Hotz-type feeding plate: printed model (occlusal view), plate in situ on the model (frontal view and occlusal view). Arrows indicate the direction of premaxillary alignment resulting from the setup simulation.

Shortly after birth, the patient with CLP is referred to an orthodontist for an intraoral optical scan using the Medit i700 scanner (Medit Corp), which offers high-resolution imaging suitable for capturing the delicate neonatal oral soft tissues. Given the challenges of moisture control in neonates, suction and cotton rolls are used to maintain a dry field during scanning. This scan enables the creation of a 3-dimensional (3D)–printed jaw model.^14^ For BCLP cases, a model is fabricated from computer-aided design data after premaxillary alignment on the software (Figure 1). During alignment, the laterally displaced premaxilla is moved 2 through 3 mm to an improved lateral position with minimal rotational correction. Anteroposterior movement is kept to a minimum.

### Fabrication of the feeding plate

Jaw models for appliance fabrication are produced via 3D printing. For this process, we used the Straumann P30+ 3D printer (Straumann Group) with a layer thickness of 50 μm. However, the choice of printer or resin is not strictly critical, and any standard resin suitable for dental modeling can be used. When printing hollow models, ensure that the wall thickness is at least 3 mm to prevent fractures during the vacuum forming process, as models with a wall thickness of 2.5 mm, for example, have fractured under molding pressure in our experience. Using the jaw model made from the above data, a 2-layer thermoplastic resin (Erkoloc-Pro; Erkodent Erich Kopp GmbH) consisting of a hard PET-G outer layer and a soft EVA inner layer with a thickness of 2.0 mm is molded using a pressure molding machine (Biostar; Scheu Dental GmbH) (Figure 1). The rigid outer layer provides structural support, while the soft inner layer protects the delicate oral mucosa of newborns. The feeding plate is designed so that the resin does not extend into the cleft spaces. To achieve optimal premaxillary positioning, selective blockout is performed during plate fabrication in the cleft regions (alveolar, palatal), on the lingual surface of the premaxilla, and on the labial surfaces of the minor segments. This blockout prevents resin from extending into undercuts and guides segment movement toward achieving a more natural maxillary morphology. Although fully digital fabrication methods are also available, creating a bilayered structure such as Erkoloc-Pro remains technically challenging, and appliances are generally produced as single-layer devices.^15^^,^^16^ Therefore, the analog vacuum-forming process continues to offer advantages in achieving a design that combines rigidity for stability and softness for mucosal protection.

### Application of PG-NAM

Traditionally, NAM appliances have incorporated acrylic protuberances attached to intraoral elastics for appliance retention, reflecting earlier protocols. However, these structures make both the fabrication process and the daily management of the appliance more complex.^10^^,^^11^ Therefore, our protocol does not use acrylic protuberances. Instead, the feeding plate is secured using highly elastic, skin-friendly medical tape (Skinergate Pitatt; Nichiban Co, Ltd), which is applied from outside the cheeks across the labial surface of the appliance. This method simplifies both fabrication and handling while maintaining sufficient retention and effective guidance of the premaxilla.

We have not encountered any cases of choking hazards with our method. However, if more secure retention is desired, the medical tape can be wrapped around the nasal stent once to provide additional stability. This technique ensures reliable appliance fixation without the need for intraoral acrylic protuberances, contributing to a simpler and safer PG-NAM protocol.

### Addition of the nasal stent

**Figure 2:**
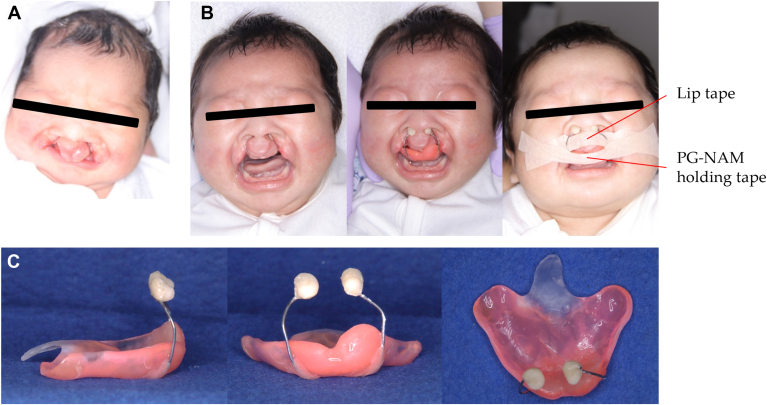
Clinical sequence of premaxilla-guiding nasoalveolar molding (PG-NAM) therapy in a patient with bilateral cleft lip and palate. **A.** Frontal facial view 1 day after application of the Hotz-type feeding plate. **B.** Sequential facial views 1 week after Hotz plate application: pre-PG-NAM (left), with PG-NAM appliance in place (center), and with lip band and PG-NAM holding band (right). **C.** Right lateral, frontal, and tissue-contacting surface views, respectively, of the PG-NAM appliance.

One week after birth, a nasal stent is applied to a Hotz plate. The stent is made from 0.8 mm stainless steel wire and self-curing resin in a beanlike shape (Figure 2).^17^ Once the stent is in place, 2 elastic lip tapes are used to prevent premaxillary collapse. The 3 steps are as follows (Figure 2):

1. Pull both lips together with the elastic lip tape.
2. Adjust the nasal stent to apply appropriate pressure to the nasal rims.
3. Secure the PG-NAM with the elastic lip tape to pull it back and up.

### Adjustments to the PG-NAM

Premaxillary alignment is typically completed within 1 through 2 months of appliance placement. Weekly visits are scheduled for nasal stent adjustments and for adding soft liner resin (Denture Soft II; Kamemizu Chemical Industry Co, Ltd) to the labial and occlusal surfaces of the feeding plate. This resin is selectively applied to exert gentle pressure and guide the premaxilla into proper alignment. To prevent reverse occlusion, care is taken not to overalign the premaxilla between the minor segments.^10^ Alignment is judged based on the position and height of the upper lip of the premaxilla. Decisions regarding lateral expansion are based on the palatal cleft width and intermolar distance at birth. Cases with a wide palatal cleft do not require lateral expansion, whereas narrow cases undergo periodic trimming every 3 weeks.

### After lip surgery

Lip surgery is performed at age 3 months, with the nasal stent removed immediately before surgery, leaving only the feeding plate. A new Hotz-type feeding plate is fabricated after surgery. As the premaxillary alignment is complete by this stage, the elastic lip tape is no longer used. The feeding plate is replaced every 3 months as needed until palatoplasty at 1 year.

In our facility, PG-NAM is applied within 4 through 24 hours of birth, with premaxillary alignment achieved in all cases by 3 months. Our report outlines the process and outcomes of 3 BCLP cases treated with this method.

### Case series

#### Case 1: Complete Bilateral Cleft Lip, Alveolus, and Palate

**Figure 3:**
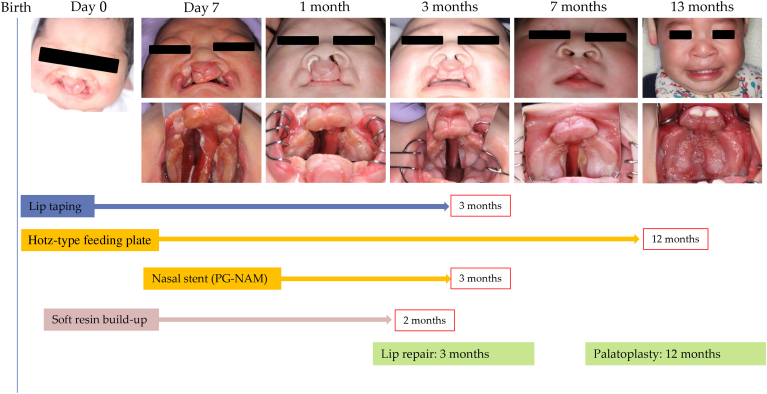
Clinical timeline of premaxilla-guiding nasoalveolar molding (PG-NAM) therapy in case 1. Sequential frontal and intraoral photographs documenting the course of PG-NAM therapy from birth through 13 months. Treatments included lip taping (until 3 months), Hotz-type feeding plate (until 12 months), PG-NAM appliance (until 3 months), and periodic labial or occlusal resin build-up (until 2 months). Primary lip repair was performed at 3 months, followed by palatoplasty at 12 months.

**Figure 4:**
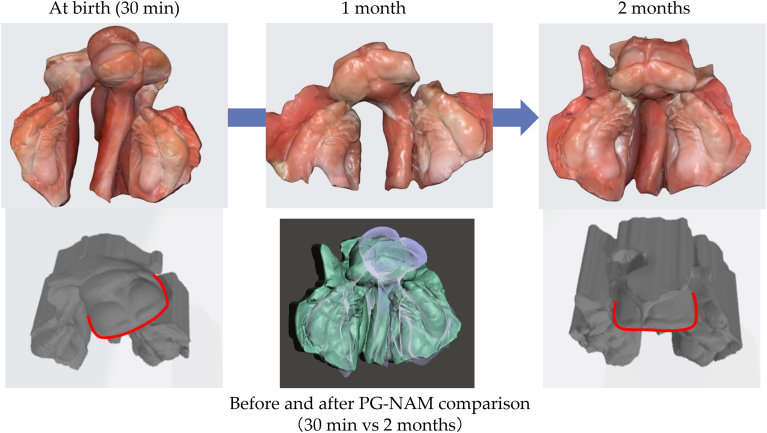
Intraoral model comparison in case 1 before and after premaxilla-guiding nasoalveolar molding (PG-NAM) therapy. Three-dimensional intraoral models showing occlusal views at birth (30 minutes), 1 month, and 2 months. The lower center image shows a superimposition of the models at birth (30 minutes) and at 2 months for comparison. The gray models in the lower panels represent upward views from the front and below, providing a supplementary perspective on the positional changes of the premaxilla segments.

A male infant was born with a complete bilateral cleft lip, alveolus, and palate (Figures 3 and 4). The premaxilla was distally rotated and intruded into the left nostril. Within 30 minutes of birth, oral impressions were obtained with an intraoral scanner while the infant was in an incubator. A simulation of the premaxillary alignment was performed on a digital model, and a feeding plate was fabricated from thermoplastic resin on a 3D-printed simulation model. The plate was fitted before the infant’s first feeding on the same day. The premaxilla achieved near midline alignment within 1 day of plate use. At 1 week of age, a nasal stent was applied, and soft resin was added to the labial and occlusal surfaces of the premaxilla. Elastic lip tape was also used to aid premaxillary alignment. PG-NAM adjustments were performed weekly by adding soft resin, while lateral expansion of the mucosal surface was executed every 3 weeks. Lip repair surgery was performed at 3 months of age, followed by palatoplasty at 12 months. The upper lip achieved sufficient length, and a normal anterior overbite was observed. The premaxilla achieved alignment without horizontal collapse. Furthermore, the deviated nasal septum, initially curved at 1 month of age, was almost straightened by 2 months of age.

#### Case 2: Complete BCLP on the Right, Incomplete Cleft Lip, and Left Alveolar and Palatal Cleft

**Figure 5:**
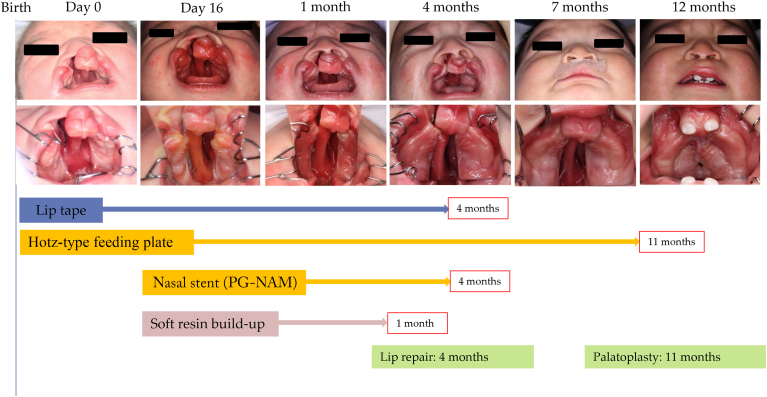
Clinical timeline of premaxilla-guiding nasoalveolar molding (PG-NAM) therapy in case 2. Sequential frontal and intraoral photographs from birth (day 0) through 12 months. Treatments included lip taping, Hotz-type feeding plate, PG-NAM appliance with nasal stent, and periodic resin build-up. Primary lip repair was performed at 4 months and palatoplasty at 11 months.

**Figure 6:**
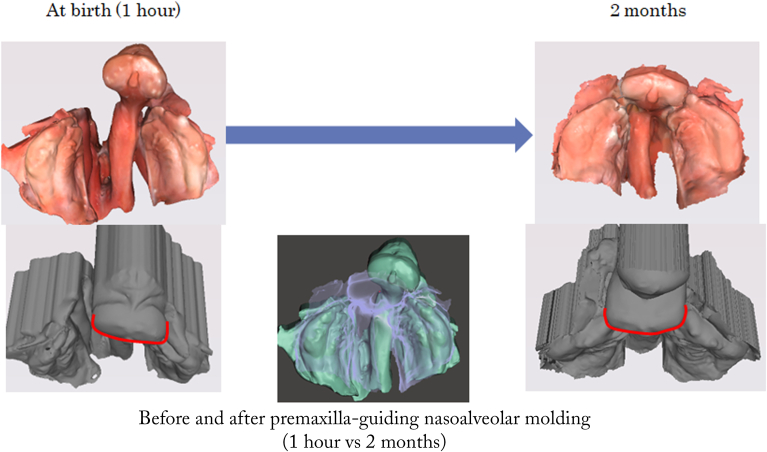
Intraoral model comparison in case 2 before and after premaxilla-guiding nasoalveolar molding therapy. Three-dimensional intraoral models showing occlusal views at birth (1 hour) and at 2 months of age. The center image shows a superimposition of the models at birth (1 hour) and at 2 months of age for comparison. The gray models in the lower panels represent upward views from the front and below, providing a supplementary perspective on the positional changes of the premaxilla segments.

A male infant was born with a forward-protruding and left-deviated premaxilla (Figures 5 and 6). Because of the nocturnal delivery, an intraoral scan was performed 16 hours after birth, followed by the fabrication of a Hotz-type feeding plate based on a premaxillary alignment model. At age 16 days, a nasal stent was added, and soft resin was applied to the labial and occlusal surfaces of the premaxilla. Two elastic lip tapes were used to begin the premaxillary alignment. Soft resin was added weekly, and premaxillary alignment was completed by age 2 months. Lip repair surgery was performed at 3 months, and the use of the Hotz-type feeding plate was continued to reduce the palatal cleft width. Lateral expansion adjustments were carried out every 3 weeks. Palatoplasty was performed at 12 months, resulting in sufficient upper lip length and a normal anterior overbite. As in the first case, the premaxilla alignment was achieved without horizontal collapse. The nasal septum, initially curved until 4 months of age, was almost straightened before palatoplasty.

#### Case 3: Complete Bilateral Cleft Lip, Alveolus, and Palate

**Figure 7:**
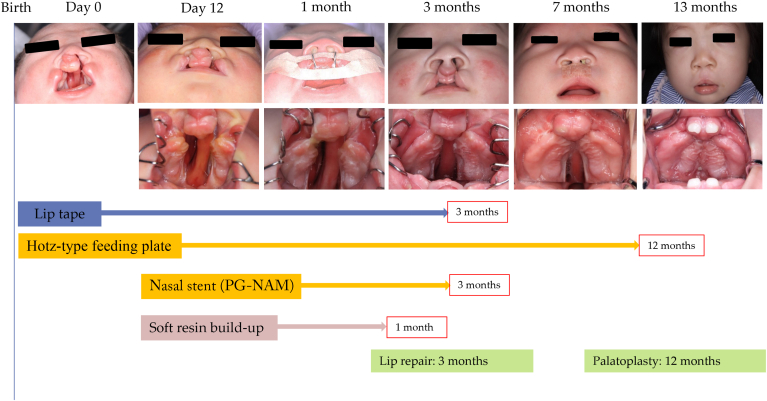
Clinical timeline of premaxilla-guiding nasoalveolar molding (PG-NAM) therapy in case 3. Sequential frontal and intraoral photographs from birth through 13 months. Treatments included lip taping, a Hotz-type feeding plate, PG-NAM appliance with nasal stent, and weekly soft resin build-up. Primary lip repair was performed at 3 months, and palatoplasty at 12 months.

**Figure 8:**
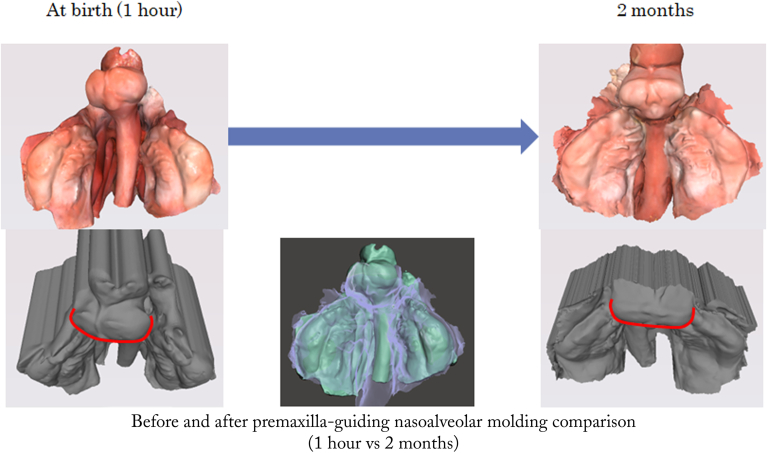
Intraoral model comparison in case 3 before and after premaxilla-guiding nasoalveolar molding therapy. Three-dimensional intraoral models showing occlusal views at birth (1 hour) and at 2 months. The center image shows a superimposition of the models at birth (1 hour) and at 2 months for comparison. The gray models in the lower panels represent upward views from the front and below, providing a supplementary perspective on the positional changes of the premaxilla segments.

A female infant was born with a complete bilateral cleft lip, alveolus, and palate (Figures 7 and 8). An intraoral scan was performed immediately after birth, and a Hotz-type feeding plate was fabricated. A nasal stent was applied at age 12 days, followed by the application of soft resin to the labial and occlusal surfaces of the premaxilla. Two elastic lip tapes were used to initiate premaxillary alignment. Weekly resin adjustments were made to achieve premaxillary alignment by 2 months. Lip repair surgery was performed at 3 months, with continued use of the Hotz-type feeding plate to reduce the width of the palatal cleft. Lateral expansion was performed every 3 weeks. Palatoplasty was conducted at 12 months, resulting in sufficient upper lip length and an adequate overbite.

## Discussion

The implementation of this method requires the collaboration of the multidisciplinary team members in a CLP center. The clinical team consists of orthodontists, pediatric dentists, oral surgeons, dental anesthesiologists, plastic surgeons, otolaryngologists, pediatricians, obstetricians, nurses, and dental technicians. In our center, the obstetricians inform the team members of the expected delivery dates for prenatally diagnosed cases. Informed consent for PG-NAM is obtained from the parents before birth, allowing ultraearly oral impressions to be taken within 30 minutes of birth. This enables early placement of the feeding plate, which facilitates rapid premaxillary alignment without collapse.

Reports indicate that nasal cartilage correction with a nasal stent is ineffective after 3 months of age.^10^ Therefore, the Hotz-type plate should be applied as early as possible after birth, followed by nasal stent placement within 1 through 2 weeks to begin premaxillary alignment and columellar lengthening.^18^ The nasal stent elevates the nostril rim, extends the columella and upper lip, and prevents the downward collapse of the premaxilla by providing upward and posterior traction via elastic lip taping. In addition, soft resin is incrementally added not only to the labial but also to the occlusal surfaces to prevent premaxillary drooping. In cases of premaxillary rotation, asymmetrical resin build-up can effectively straighten the premaxilla.^19^

Our approach differs from conventional methods, which typically delay obtaining impressions and fabricating feeding plates for several days through weeks after birth.^12^^,^^13^ Such delays may hinder spontaneous cleft narrowing and complicate early premaxillary alignment. Moreover, Grayson and Cutting^12^ noted that earlier appliance insertion improves infant acceptance and molding efficiency, suggesting that delayed use may prolong adaptation and hinder consistent wearing.^10^ In contrast, our ultraearly protocol, initiating treatment within 24 hours of birth (as early as 4 hours), allows for immediate intervention, promotes early premaxillary repositioning, and helps prevent deformation. This early start may also enhance patient compliance and improve overall therapeutic outcomes.

Previous studies have further supported the importance of timing in NAM therapy.^20^^,^^21^ Shetty et al^22^ compared the outcomes of early vs delayed initiation of NAM in infants with unilateral CLP (UCLP). They reported that starting NAM before 1 month of age led to significantly greater improvements in nasal and alveolar morphology than later initiation. Similarly, Park et al^20^ found that after age 4 weeks, patients were significantly less likely to undergo NAM because of concerns about the diminished effectiveness of nasal cartilage molding. Although these studies focused on UCLP, their findings align with our observations. In our report, all 3 cases, each diagnosed with UCLP, achieved favorable nasal and premaxillary alignment by age 3 months following ultraearly PG-NAM initiated within 24 hours of birth, and in some cases as early as 4 hours postpartum. Although Shetty et al^22^ defined early as initiation within the first month of life, our protocol started even earlier, within 24 hours postpartum. Such ultraearly intervention may offer additional benefits by maximizing the molding potential of neonatal cartilage before the progression of cleft-related deformities. Our results suggest that the therapeutic window for optimal nasal and premaxillary remodeling may be narrower than previously believed. Although further comparative studies are needed to clarify whether ultraearly initiation indeed offers superior outcomes, data regarding BCLP remain limited, emphasizing the need for multicenter studies in this area.

In our method, a feeding plate is fabricated during the initial PG-NAM process using a digitally simulated premaxillary alignment model. This facilitates rapid midline alignment of the premaxilla. Once midline alignment is achieved, posterior movement of the premaxilla may be initiated for faster overall alignment. Because of the significant lateral mobility of the premaxilla in cases with BCLP, lateral movement of approximately 2 through 3 mm can be achieved immediately. However, anteroposterior movement of the premaxilla requires attention, as excessive mucosal pressure may lead to alveolar mucosal ulceration. Gradual adjustments with soft resin are essential.

Massie et al^21^ showed significant septal deviation in skeletally mature CLP patients compared with control patients. Similarly, Starbuck et al^23^ and Holton et al^24^ found that nasal septal deviation and associated facial asymmetries were common in CLP patients, suggesting that conventional surgical approaches may not adequately address these deformities. In all 3 cases in our study, nasal septal curvature was observed after posterior premaxillary movement. However, at the time of palatoplasty, the nasal septum was straightened in all cases. Several studies have reported persistent nasal septal deviation in patients with BCLP who underwent conventional palatoplasty without early orthopedic management. These findings support the potential benefit of ultraearly intervention, such as PG-NAM, in improving nasal septal alignment. This suggests that nasal septal straightening may occur without direct control using a Hotz-type plate that extends into the nasal cavity. In our clinical experience, ultraearly initiation of NAM treatment immediately after birth appears to achieve faster and more favorable premaxillary alignment and earlier columellar lengthening than our previous cases, in which the Hotz plate was introduced at approximately age 2 weeks.

In many cases of ultraearly initiation, presurgical orthopedic treatment is completed by age 2 months. However, our number of cases remains limited, and we have not yet conducted objective evaluations using facial photographs or digital model measurements. Further studies with larger sample sizes and objective assessment methods are necessary to validate these preliminary observations. Nevertheless, the ultraearly application of PG-NAM might be a feasible remedy to achieve stable premaxillary alignment.

## Conclusions

We presented 3 cases of BCLP where early application of PG-NAM achieved proper premaxillary alignment, columella and upper lip lengthening by 2 months, allowing for smooth surgical interventions. Premaxillary alignment was achieved at 2 months. Although 3 cases involved nasal septal curvature after posterior premaxillary movement, the nasal septum was straightened at the time of palatoplasty. Our findings suggest the potential for nasal septal straightening to occur without direct control using a Hotz-type plate that extends into the nasal cavity. However, further research is necessary to validate this observation. Taken together, the ultraearly application of PG-NAM might be a feasible remedy to achieve stable premaxillary alignment.

## Disclosure

None of the authors reported any disclosures.
